# PD-L1 expression in epithelial ovarian cancer and survival: a retrospective cohort study

**DOI:** 10.61622/rbgo/2026rbgo731

**Published:** 2026-10-30

**Authors:** Lucas Dorídio Locks-Coelho, Charles Francisco Ferreira, Edison Capp, Márcia Luiza Appel, Lúcia Maria Kliemann

**Affiliations:** 1 Universidade Federal do Rio Grande do Sul Porto Alegre RS Brazil Universidade Federal do Rio Grande do Sul, Porto Alegre, RS, Brazil.; 2 Hospital de Clínicas de Porto Alegre Porto Alegre RS Brazil Hospital de Clínicas de Porto Alegre, Porto Alegre, RS, Brazil.

**Keywords:** Ovarian neoplasms, Programmed cell death 1 receptor, Survival analysis, Tumor microenvironment

## Abstract

**Objective:**

To evaluate PD-L1 expression, measured by the Combined Positive Score (CPS), and its association with overall survival (OS) and progression-free survival (PFS) in patients with epithelial ovarian cancer (EOC).

**Methods:**

This retrospective cohort study included patients with EOC from southern Brazil. PD-L1 immunostaining was performed on tissue microarrays, and expression in tumor and inflammatory cells was assessed using CPS. Patients were classified as PD-L1 positive (CPS ≥1) or negative. Associations with clinicopathological variables, OS, and PFS were analyzed. Kaplan-Meier curves with log-rank testing were used for the primary time-to-event comparisons, and multivariable Cox regression was performed to assess independent prognostic associations.

**Results:**

Among 75 patients, 21(28%) were PD-L1 positive and 54(72%) were negative. Clinicopathological characteristics did not differ significantly between groups. Follow-up ranged from 0 to 185 months. In descriptive analyses, PD-L1-positive patients had longer median OS (66 vs. 36.5 months) and PFS (38 vs. 20.5 months). However, Kaplan-Meier analysis with log-rank testing showed no statistically significant differences in OS or PFS between groups. In multivariable Cox regression, PD-L1 positivity was independently associated with a lower risk of death (adjusted HR 0.48, 95% CI 0.24-0.99; p = 0.047), but not with recurrence.

**Conclusion:**

PD-L1 positivity was associated with a lower risk of death in adjusted analysis, although no significant differences were observed in Kaplan-Meier survival curves. These findings should be interpreted cautiously and considered exploratory. Larger prospective multicenter studies with standardized PD-L1 assessment are needed to validate these results.

## Introduction

Epithelial ovarian cancer (EOC) is one of the most common malignancies and represents the second leading cause of death among gynecologic cancers.^(1,2)^ In Brazil, EOC accounted for more than 7,000 new cases in 2022 and over 4,000 deaths in the preceding year, reflecting a concerning similarity between incidence and mortality rates per 100,000 inhabitants, as observed worldwide.^(1,3)^ Most patients are diagnosed at advanced stages (FIGO stages III and IV), largely due to the lack of effective screening tools and nonspecific early clinical symptoms, with reported 5-year median survival rates ranging from 30% to 50%.^(4,5)^ Despite standard treatment with cytoreductive surgery combined with platinum- and taxane-based chemotherapy, mortality rates remains high. Therefore, new approaches to better understand the disease, refine prognostic assessment, and develop more effective therapeutic strategies are urgently needed.

Many new drugs have emerged and demonstrated beneficial effects in various clinical settings, including adjuvant therapy, maintenance, and recurrent disease. Examples include bevacizumab, an antiangiogenic agent that inhibits vascular endothelial growth factor (VEGF), and poly (ADP-ribose) polymerase (PARP) inhibitors, both of which already incorporated into first-line treatment strategies.^(6–9)^ Another promising therapeutic class is immunotherapy, particularly immune checkpoint inhibitors (ICIs), which are currently being developed and evaluated across multiple cancer types.^(10,11)^ Increasing attention has been directed toward the tumor microenvironment and its immunologic interface,^(12)^ underscoring the importance of understanding these mechanisms to elucidate tumor pathogenesis and identify novel therapeutic targets.

Programmed cell death ligand 1 (PD-L1), a immune-related transmembrane biomarker expressed in several malignant tumors, including EOC, serves as an important indicator of prognosis and eligibility for targeted therapies.^(11–13)^ PD-L1 exerts its function primarily by binding to its receptor, PD-1 (programmed cell death protein 1), which is expressed mainly on immune cells. This interaction downregulates immune responses by inducing apoptosis in activated T cells, suppressing cytokine production, and inhibiting T-cell proliferation, thereby enabling tumor cells to evade immune surveillance.^(13,14)^ Interest in the prognostic significance of PD-L1 expression has grown substantially,^(13,15–17)^ alongside the expanding clinical application of therapies targeting this pathway.^(11,18)^

However, the precise prognostic implications of PD-L1 expression in EOC remain unclear, with studies reporting conflicting results.^(13,15–17,19–23)^ Even meta-analyses have yielded inconsistent findings, largely due to the pooling of data from different histological subtypes, small sample sizes, and variability in scoring methods and cut-off values.^(24–28)^ The Combined Positive Score (CPS) is one such scoring system used to assess PD-L1 expression by immunohistochemistry (IHC) in surgical specimens. CPS is calculated as the total number of PD-L1–positive tumor cells and inflammatory cells (including tumor-infiltrating lymphocytes, TILs) divided by the total number of viable tumor cells, multiplied by 100.^(23)^ This comprehensive metric, which accounts for both tumor and immune cells, is well established and widely applied in clinical practice to determine eligibility for targeted immunotherapy across various cancer types.^(18,29,30)^

In this study, we performed PD-L1 immunostaining on tissue microarrays from EOC specimens to evaluate its expression in tumor and inflammatory cells using CPS. We further assessed its impact on overall survival, progression-free survival, and its association with other prognostic factors.

## Methods

### Patients and variables

Patients with primary EOC, treated by the Gynecologic Oncology Team at *Hospital de Clínicas de Porto Alegre* (Porto Alegre, Rio Grande do Sul, Brazil) between 2007 and 2019, were included in this retrospective cohort. Patients were excluded if they had a borderline tumor diagnosis (n = 63), an uncertain diagnosis (n=9), another synchronous malignancy (n = 5), or unavailable tissue blocks (n = 29). The biospecimens and data for this study were provided by the Pathology Service of the Hospital de Clínicas de Porto Alegre. All cases were evaluated by direct microscopic examination of hematoxylin- and eosin-stained, formalin-fixed, paraffin-embedded surgical specimens. Pathologic data, including histologic subtype, tumor grade, and tumor stage, were obtained from the original pathology reports. A retrospective review of electronic medical records provided clinical information such as residual disease (degree of debulking), chemotherapy response, recurrence, and death. Histologic subtype and grade were classified according to World Health Organization (WHO) criteria,^(31)^ and tumor stage was determined according to the International Federation of Gynecology and Obstetrics (FIGO) classification.^(4)^ For Cox regression analyses, tumor stage was reclassified as early (stages I-II) or advanced (stages III-IV). Overall survival (OS), the primary endpoint, was defined as the time from treatment initiation (surgery or chemotherapy) to death from any cause or last follow-up. Progression-free survival (PFS), the secondary endpoint, was defined as the time from treatment initiation to the first documented evidence of disease progression or recurrence.

### Tissue microarray and immunohistochemistry

Under microscopic examination, two morphologically representative tumor areas were selected and annotated on hematoxylin- and eosin-stained slides for each patient. Corresponding 2-mm tissue cores were obtained from the same areas in formalin-fixed, paraffin-embedded blocks, as marked on the hematoxylin- and eosin-stained slides, and arrayed into recipient paraffin blocks. Each tissue microarray (TMA) block contained 60 cores, four of which were replaced with positive controls (placental tissue). TMA blocks were sectioned at 3-μm thickness, and immunohistochemical (IHC) staining for PD-L1 was performed on TMA sections using the BenchMark ULTRA automated staining platform (Ventana Medical Systems, Tucson, AZ) with an anti-PD-L1 primary antibody (clone SP263, Ventana Roche, Tucson, AZ). Deparaffinization was carried out on the instrument using EZ PREP reagent. Antigen retrieval was performed with CC1 buffer (pH 9.0) at 100 °C for 92 minutes, followed by peroxidase blocking with the OptiView Peroxidase Inhibitor reagent (3% H_2_O_2_) included in the detection system. The primary anti-PD-L1 antibody (clone SP263) was incubated for 16 minutes at 36 °C. Detection was performed using the OptiView HRP Multimer system with diaminobenzidine (DAB) as the chromogen. Slides were counterstained with Mayer's hematoxylin, differentiated with a bluing reagent (Li_2_CO_3_ + Na_2_CO_3_), and examined after dehydration and mounting. These TMA blocks and IHC slides were prepared at the Laboratory Research Unit of the Experimental Research Center and the Department of Pathology, both at the Hospital de Clínicas de Porto Alegre, following established protocols.

### Immunohistochemical analysis

Tumor cells and inflammatory cells (tumor-infiltrating lymphocytes and macrophages) were analyzed, and both were considered immunopositive if either cytoplasmic or membranous staining was present. For PD-L1, the Combined Positive Score (CPS) was calculated as the total number of tumor and inflammatory cells positive for PD-L1 on IHC, divided by the total number of viable tumor cells and multiplied by 100.^(23)^ CPS assessment was performed by a certified pathologist with expertise in the field, who was completely blinded to the case identity and duplicate cores. The final CPS for each case was calculated as the mean of the two tumor areas analyzed. The cohort was divided into two groups according to PD-L1 status, using a CPS cutoff of ≥ 1 versus < 1, as previously applied in several studies.^(23,32–34)^

### Statistical analysis

Continuous variables were described as mean ± standard deviation (SD) or median with interquartile range (IQR; 25th-75th percentiles), according to data distribution assessed by the Shapiro–Wilk test. Categorical variables were expressed as absolute counts (n) and relative frequencies (%). Differences between the two groups were analyzed using Student's t-test for independent samples, the Mann-Whitney U test, or the Chi-square test with adjusted residual analysis, as appropriate. The agreement of IHC expression values between duplicate cores was assessed using the Kappa statistic. Overall survival (OS) and progression-free survival (PFS) were analyzed using the Kaplan-Meier method, with the log-rank test applied for curve comparison. The impact of each prognostic factor (including the variable under study) on OS and PFS was evaluated using bivariate and multivariate Cox regression analyses. Kaplan–Meier/log-rank and Cox regression analyses were considered the primary inferential methods for survival analysis. Median survival values were reported for descriptive purposes only. In the multivariate model, in addition to the variable under investigation, all variables with p < 0.20 in the bivariate analysis were included to control for confounding. A two-tailed p-value < 0.05 was considered statistically significant. Statistical analyses were performed using SPSS Statistics for Windows, version 21.0 (SPSS Inc., Chicago, IL, USA). The datasets generated and/or analyzed during the current study are available from the corresponding author upon reasonable request.

The study was conducted in accordance with the Declaration of Helsinki and approved by the Research Ethics Committee of *Hospital de Clínicas de Porto Alegre* 4.692.269 (CAAE no 44506621.3.0000.5327), Porto Alegre, Rio Grande do Sul, Brazil.

## Results

Table 1 presents the descriptive characteristics of the two groups, stratified by CPS cutoff, along with clinicopathologic data, including age, baseline CA-125, type of surgery, surgical stage, residual tumor (debulking status), tumor grade, histologic subtype, chemotherapy response, and survival outcomes (progression-free and overall).

**Table 1:** Descriptive analysis from cohort according to the PD-L1 expression measured through CPS and difference between groups

Variables		Total (n=75)	Combined Positive Score (CPS)		*p-value
			CPS < 1 (n=54)	CPS 1 (n=21)	
Age		57.3 ± 13.6	57.4 ± 14.2	57.0 ± 12.0	0.389
Initial CA 125^a^		578.1[109.4 - 1548.0]	735.3 [134.6 – 1737.6]	533.2 [94.0 - 1252.0]	0.721
Surgery^b^					0.639
	Primary	63(86.3)	46(88.5)	17(81.0)	
	Interval	10(13.7)	6(11.5)	4(19.0)	
Staging (FIGO)					0.084
	I	20(26.7)	17(31.5)	3(14.3)	
	II	4(5.3)	1(1.9)	3(14.3)	
	III	45(60.0)	31(57.4)	14(66.7)	
	IV	6(8.0)	5(9.3)	1(4.8)	
Histologic subtype^c^					0.499
	Serous	42(58.3)	29(56.9)	13(61.9)	
	Mucinous	5(6.9)	5(9.8)	0(0.0)	
	Endometrioid	11(15.3)	8(15.7)	3(14.3)	
	Clear cells	9(12.5)	5(9.8)	4(19.0)	
	Others	5(6.9)	4(7.8)	1(4.8)	
Histologic grade					0.126
	Low grade	18(24.0)	16(29.6)	2(9.5)	
	High grade	57(76.0)	38(70.4)	19(90.5)	
Debulking^d^					0.614
	Complete	36(48.6)	24(45.3)	12(57.1)	
	Optimal (1 cm)	19(25.7)	15(28.3)	4(19.0)	
	Suboptimal (> 1 cm)	19(25.7)	14(26.4)	5(23.8)	
Recurrence^e^					0.145
	Platinum sensitive	48(87.3)	31(81.6)	17(100.0)	
	Platinum resistant	7(12.7)	7(18.4)	0(0.0)	
	PFS (months)^f^	22.0 [12.0 - 65.5]	20.5 [8.0 - 61.5]	38.0 [15.0 – 82.5]	0.048
	OS (months)	51.0 [17.5 - 75.0]	36.5 [15.0 - 67.0]	66.0 [50.0 – 87.0]	0.014

The data are presented as means ± SD, medians [IQR], or absolute (n) and relative frequencies (%). Comparisons between groups were performed using Student's t-test for independent samples, the Mann-Whitney test, or the Chi-square test with adjusted residual analyses. Legend: CPS - Combined Positive Score; FIGO - International Federation of Gynecology and Obstetrics; PFS - progression-free survival; OS - overall survival. Significance was set at 5% for all analyses.

a <1 n = 48(87.3%), ≥1 n = 20(95.2%)

b <1 n = 52(94.5%), ≥1 n = 21(100.0%)

c <1 n = 51(92.7%), ≥1 n = 21(100.0%)

d <1 n = 53(96.4%), ≥1 n = 21(100.0%)

e <1 n = 38(69.1%), ≥1 n = 17(80.9%)

f <1 n = 52(94.5%), ≥1 n = 20(95.2%)

Of the 75 patients, 21(28%) had CPS ≥ 1 and 54(72%) had CPS < 1. No significant differences were observed between the groups in clinicopathologic characteristics, including age, baseline CA-125, type of surgery, surgical staging, residual tumor status, histologic subtype, tumor grade, and chemotherapy response (platinum sensitivity). Patients’ ages ranged from 19 to 88 years, with a mean age of 57.0 ± 12.0 years in the CPS ≥ 1 group and 57.4 ± 14.2 years in the CPS < 1 group (p = 0.389). A total of 63 patients underwent primary surgery, whereas 10 underwent interval surgery, with no difference in PD-L1 expression between groups (p = 0.639), indicating no variation in CPS when biospecimens collected before versus after neoadjuvant chemotherapy were analyzed. As expected, most cases were advanced (FIGO stage III or IV), totaling 51(68%) cases. Serous histology was observed in 42(58.3%) patients, while 30(41.7%) had non-serous histology, including mucinous (n = 5), endometrioid (n = 11), clear cell adenocarcinoma (n = 9), and other types (n = 5, most of which were undifferentiated). Regarding histologic grade, among patients with CPS ≥ 1, two cases were low grade and 19 were high grade; in the CPS < 1 group, 16 were low grade and 38 were high grade, with no statistically significant difference (p = 0.126). Representative cases with corresponding CPS values are shown in figure 1.

**Figure 1:**
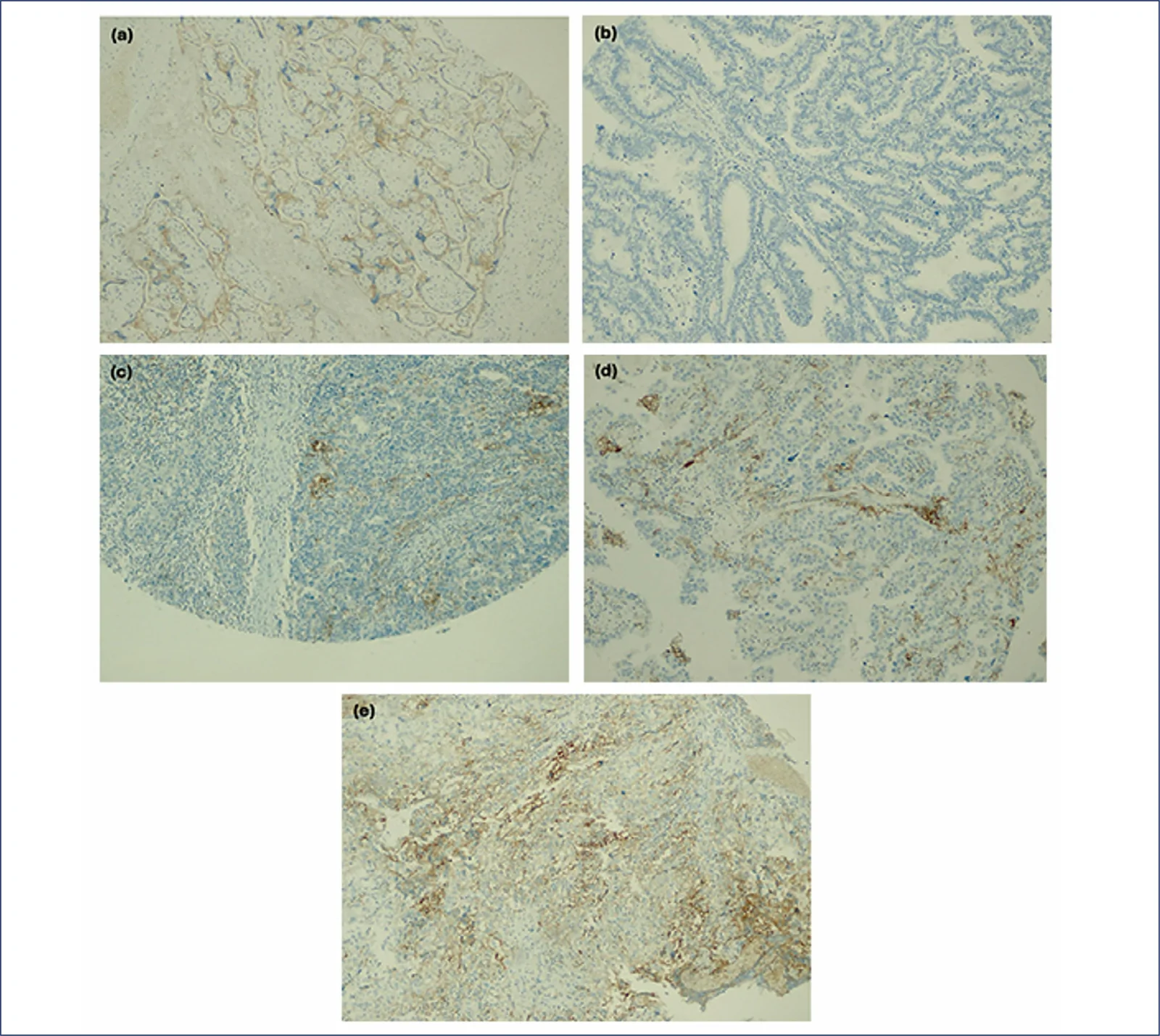
Representative cases of PD-L1 expression according CPS (combined positive score)

Images with magnification ×200; (a) Positive control - placenta, (b) CPS = 0, (c) CPS = 3, (d) CPS = 40, (e) CPS = 80.

Most patients (n=63) underwent primary cytoreductive surgery followed by six cycles of adjuvant carboplatin- and paclitaxel-based chemotherapy. Ten patients underwent interval debulking after three cycles of neoadjuvant chemotherapy (NACT), followed by three additional adjuvant cycles, while two patients underwent biopsy only and subsequently received chemotherapy. Regarding the degree of cytoreduction, 36 cases(48.6%) achieved complete debulking (no macroscopic disease), 19(25.7%) achieved optimal debulking (residual disease ≤ 1 cm), and 19(25.7%) had suboptimal debulking (residual disease > 1 cm), with no significant difference in PD-L1 expression between groups (p = 0.614). Among the 55 patients (73.3%) who experienced recurrence, seven(12.5%) relapsed within 6 months after completing adjuvant chemotherapy (platinum-resistant), whereas 48(87.3%) were platinum-sensitive. No significant difference was observed between the CPS groups (p = 0.145). Kaplan–Meier analysis showed no statistically significant differences between the PD-L1 groups for progression-free survival (log-rank p = 0.643) or overall survival (log-rank p = 0.126) (Figure 2). The median PFS was 38 months in patients with CPS ≥ 1 and 20.5 months in those with CPS < 1, while the median OS was 66 and 36.5 months, respectively. These median values are presented descriptively.

**Figure 2:**
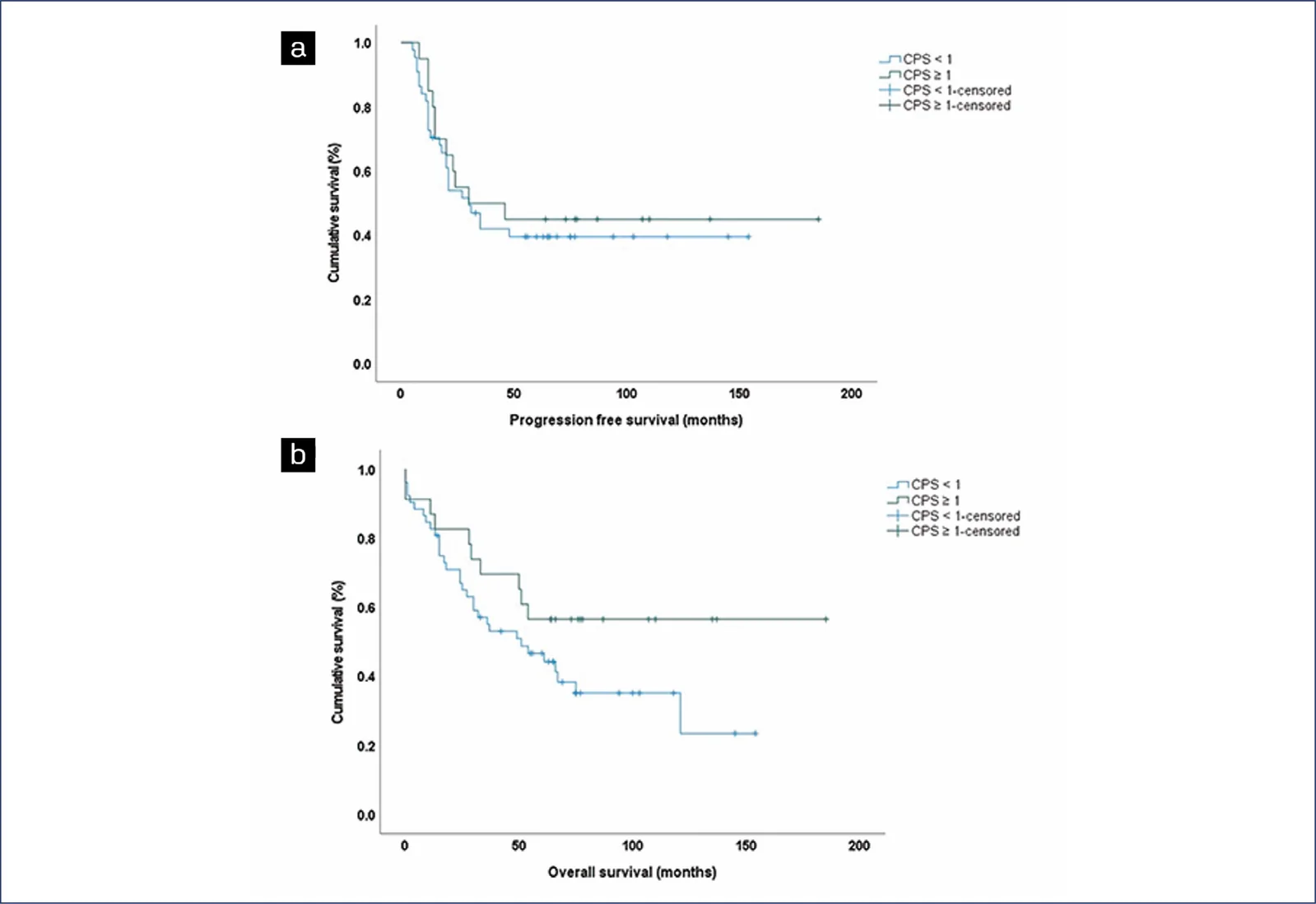
Kaplan-Meier curves and log-rank tests for the cohort according to PD-L1 expression (Combined Positive Score [CPS] ≥ 1 vs. < 1) in ovarian cancer

(a) Progression-free survival (log-rank p = 0.643); (b) Overall survival (log-rank p = 0.126).

In bivariate Cox regression analysis for recurrence risk (Table 2), CPS showed no significant association (unadjusted HR 0.85, 95% CI 0.42–1.72, p = 0.649). In contrast, advanced surgical stage (III–IV), suboptimal debulking, and high histologic grade were associated with increased risk, with unadjusted HRs of 5.89(95% CI 2.45–14.19, p ≤ 0.001), 3.90(95% CI 1.67–9.08, p = 0.002), and 4.85(95% CI 1.72–13.71, p = 0.003), respectively. In the multivariate Cox regression analysis, only advanced stage remained an independent risk factor (adjusted HR 6.88, 95% CI 2.64–17.90, p ≤ 0.001).

**Table 2:** Factors associated with recurrence in ovarian cancer in the bivariate and multivariate analysis (Cox regression)

Variables	Unadjusted		Adjusted	
	HR(95% CI)	*p-value	HR(95% CI)	*p- value
CPS 1	0.85 (0.42-1.72)	0.649	0.61 (0.29-1.29)	0.199
Staging FIGO III+IV	5.89 (2.45-14.19)	≤0.001	6.88 (2.64-17.90)	≤0.001
Suboptimal debulking	3.90 (1.67-9.08)	0.002	1.43 (0.53-3.88)	0.480
High grade histology	4.85 (1.72-13.71)	0.003	2.26 (0.58-8.87)	0.240

Data presented as Hazard Ratio (HR) with 95% confidence interval (CI) by

* Cox regression. CPS - Combined Positive Score; FIGO - International Federation of Gynecology and Obstetrics; p - Statistical significance index. Significance level set at 5% for all analyses

In the multivariate model (Table 3), after adjustment for surgical stage, debulking status, and tumor grade, PD-L1 expression (CPS ≥ 1) emerged as an independent protective factor (adjusted HR 0.48, 95% CI 0.24–0.99, p = 0.047), whereas advanced surgical stage remained an independent risk factor (adjusted HR 5.05, 95% CI 2.11–12.08, p ≤ 0.001).

**Table 3:** Factors associated with death in ovarian cancer in the bivariate and multivariate analysis (Cox regression)

Variables	Unadjusted		Adjusted	
	HR(95% CI)	*p-value	HR(95% CI)	*p-value
CPS 1	0.58 (0.28-1.18)	0.133	0.48 (0.24-0.99)	0.047
Staging FIGO III+IV	4.82 (2.03-11.44)	0.027	5.05 (2.11-12.08)	≤0.001
Suboptimal debulking	3.91 (1.87-8.19)	≤0.001	2.05 (0.87-4.84)	0.100
High grade histology	3.41 (1.34-8.66)	0.010	1.88 (0.60-5.88)	0.280

Data presented as Hazard Ratio (HR) with 95% confidence interval (CI) by

* Cox regression. CPS - Combined Positive Score; FIGO - International Federation of Gynecology and Obstetrics; p - Statistical significance index. Significance level set at 5% for all analyses

Agreement between the CPS values from the two tumor areas, assessed by the kappa statistic, demonstrated moderate concordance (κ = 0.597).

## Discussion

In this retrospective cohort study of 75 patients with epithelial ovarian cancer, 28% of tumors were PD-L1 positive (CPS ≥ 1). PD-L1 expression was not associated with clinicopathologic characteristics, including age, stage, histologic subtype, grade, debulking status, or platinum sensitivity. Patients with PD-L1-positive tumors showed longer descriptive median progression-free survival and overall survival than PD-L1–negative patients. However, Kaplan–Meier analysis with log-rank testing did not demonstrate statistically significant differences in survival distributions between groups. Therefore, the median differences should be interpreted as secondary descriptive findings rather than as primary inferential evidence.

Overall, 28% of patients with ovarian cancer were PD-L1–positive (CPS ≥ 1). This prevalence falls within the wide range reported in the literature(12%–80%), which is largely attributable to methodological differences, including the antibody clone used, scoring systems applied, and cutoff definitions.^(24–28)^ Studies employing the same antibody clone as ours (SP263) have demonstrated positivity rates ranging 12% to 64%.^(16,20,22,35)^ In contrast, investigations using CPS with the same cutoff but different antibodies have reported rates between 37% and 60%.^(32–34)^ These discrepancies underscore the need for standardization in PD-L1 assessment, as methodological variability may obscure its prognostic significance in EOC and complicate cross-study comparisons.

No significant differences were observed between the cohort groups in terms of clinicopathologic characteristics, such as age, baseline CA 125, type of surgery, stage, residual tumor, histologic subtype, histologic grade, and response to chemotherapy (platinum sensitivity). Regarding histologic subtype, several studies have reported higher PD-L1 positivity in serous than in non-serous tumors;^(15,19,21)^ however, this finding was not reproduced in our cohort, even when comparing serous tumors(58.3% of the sample) to all non-serous cases combined (p=0.696, data not shown). With respect to histologic grade, although the difference was not statistically significant (p=0.126), 16 of 18 low-grade cases showed no PD-L1 expression. Similarly, platinum sensitivity among recurrences did not differ between groups (p=0.145), although none of the seven platinum-resistant cases were PD-L1 positive. These trends may be explained by the relatively small sample size and are consistent with some reports associating PD-L1 expression with aggressive histology, advanced stage, and platinum-sensitive recurrence.^(13,23)^ Conversely, a 2019 meta-analysis found no association with any of these clinicopathological features,^(26)^ whereas a 2023 meta-analysis reported higher PD-L1 expression only in the high-grade serous subtype.^(28)^

No difference in PD-L1 expression was observed between samples obtained before or after chemotherapy. This comparison included specimens from primary and interval surgery. These findings contrast with some previous studies and may be explained by the small number of patients in the interval surgery subgroup. Neoadjuvant chemotherapy (NACT) was considered due to its known influence on TILs and PD-L1 expression in ovarian cancers: Mesnage et al. demonstrated increased PD-L1 expression in tumor cells and tumor-infiltrating lymphocytes after NACT;^(36)^ similar findings were reported Lee and colleagues,^(37)^ and by Chen in tumor cells only.^(32)^

The prognostic role of PD-L1 in EOC remains uncertain, largely due to conflicting evidence in the literature. In the present study, PD-L1-positive patients showed longer descriptive median PFS and OS than PD-L1-negative patients. However, Kaplan–Meier survival curves did not demonstrate statistically significant differences between groups for either endpoint. Accordingly, these median differences should not be interpreted as clinically definitive evidence of superior survival, particularly given the presence of right-censoring and the limited sample size. The multivariate Cox model suggested an association between PD-L1 positivity and lower mortality risk after adjustment for prognostic factors, but this finding should be interpreted cautiously and considered exploratory.

It should be noted that PD-L1 expression was not statistically significant in the unadjusted Cox model but became significant after multivariate adjustment. This finding likely reflects negative confounding by strong prognostic variables, such as disease stage and residual tumor, which were included in the final model. Adjustment for these factors reduced residual variance and strengthened the association between PD-L1 expression and overall survival. Nevertheless, given the modest sample size and limited number of PD-L1-positive cases, this result should be interpreted cautiously, as limited precision of the estimates and residual confounding cannot be excluded.

In line with these findings, a positive prognostic effect of PD-L1 expression (in tumor and/or inflammatory cells) on OS was also reported by Høgdall et al., who analyzed 376 patients across all EOC subtypes using CPS (adjusted HR 0.72, 95% CI 0.56–0.93).^(23)^ Other studies have similarly demonstrated favorable prognostic associations, including one involving 288 patients with EOC^(38)^, another involving 215 patients with high-grade serous ovarian cancer by Darb-Esfahani et al.^(19)^, and a subgroup analysis of 195 patients with high-grade serous histology, particularly when PD-L1 was expressed in macrophages.^(15)^ Kim et al., using the same SP263 antibody in 246 patients, also observed improved OS when PD-L1 was expressed in stromal TILs, especially in serous carcinoma, wheras no association was found between PD-L1 expression in tumor cells and prognosis.^(16)^

In contrast, PD-L1 expression has been associated with worse overall survival in some studies, most of which defined PD-L1 positivity based on high expression in tumor cells. These include cohorts of 70 patients with EOC,^(13)^ 112 patients predominantly with serous carcinoma,^(39)^ and 122 patients with clear cell ovarian adenocarcinoma.^(21)^

A combined analysis of PD-L1 expression in tumor cells and CD8+ TILs, reported in a systematic review, suggested prognostic stratification: patients with negative tumor PD-L1 expression and a high density of CD8+ TILs had the longest median OS, whereas those with positive tumor PD-L1 expression and a low density of CD8+ TILs had the poorest survival.^(40)^

Regarding the use of CPS in EOC, apart from the aforementioned study by Høgdall et al.,^(23)^ which reported findings similar to the present study, other investigations have been inconclusive. Bansal et al. found no difference between CPS and TPS (tumor proportion score, assessing only tumor cells) for PD-L1 expression in relation to PFS in a cohort of 100 cases.^(41)^ Similarly, Chen et al. reported no association between CPS and PFS; however, PD-L1 expression assessed by TPS in high-grade serous carcinoma was associated with improved OS and PFS, both at a cutoff of 1, in 189 patients.^(32)^ Other studies have also reported no significant association between PD-L1 expression and survival outcomes.^(33,34)^

The heterogeneity of results across studies may be attributed to differences in antibody clones, scoring systems used to assess PD-L1 expression, cutoff points for positivity, and cellular targets (tumor cells and/or TILs), as well as ethnic differences and baseline characteristics. These conflicting findings highlight the need for a standardized approach to PD-L1 assessment and to establish its prognostic value in EOC. Two recent meta-analyses that included a greater number of small and negative studies found no overall association between PD-L1 expression and survival outcomes in the pooled data. However, subgroup analyses suggested higher PD-L1 expression in high-grade serous carcinoma compared with less common EOC subtypes, and an association with better OS in this subtype, particularly among Caucasian patients, while indicating a poorer prognosis in Asian populations.^(24,25)^ Conversely, Piao et al. reported the opposite in a meta-analysis with high heterogeneity, identifying PD-L1 expression as a negative predictive factor in non-Asians, but still showing a favorable prognosis in serous carcinoma and a poorer prognosis in non-serous subtypes.^(27)^ Similarly, Wang's meta-analysis of 20 studies concluded that PD-L1 expression by IHC may not be prognostic; however, bioinformatic analysis of mRNA expression revealed an association with shorter PFS.^(26)^

To our knowledge, this is the first Brazilian study to evaluate PD-L1 expression in EOC with survival analysis, contributing to the understanding of potential population-related differences reported elsewhere. Geographic region is relevant, as genetic and environmental factors may influence prognosis.^(28)^ The association between higher PD-L1 expression and better prognosis may relate to the tumor microenvironment, particularly inflammatory cells (TILs and macrophages). TILs are key mediators of the immune response, and PD-L1 expression may reflect an adaptive immune reaction to tumor growth, resulting in a favorable "immunoinflammatory" or "hot" tumor phenotype – characterized by CD4+/CD8+ infiltration, PD-L1 expression, and high levels of pro-inflammatory cytokine levels.^(19,28,42)^ This adaptive immunity may be mediated by interferon gamma and involve receptors such as PD-1, CTLA-4, LAG-3, and TIM-3, potentially broadening the benefit from immune checkpoint inhibitors.^(43–45)^

This study has some limitations. The relatively small sample size, particularly in the PD-L1–positive subgroup (n = 21), limits the statistical power to identify independent prognostic factors and reduces the precision of hazard ratio estimates. In addition, the wide range of follow-up times and the presence of right-censored observations limit the interpretability of median-based survival comparisons, which should be regarded as descriptive rather than inferential. The retrospective observational design is also inherently subject to selection bias, missing data, and residual confounding, despite multivariable adjustment. Furthermore, as this was a single-center study, the generalizability of the findings may be limited. In this context, the multivariable results should be interpreted with caution, and the observed association between PD-L1 expression and survival should be considered exploratory. Larger prospective multicenter studies with standardized PD-L1 assessment are needed to validate these findings.

## Conclusion

To our knowledge, this is the first Brazilian study to evaluate PD-L1 expression in ovarian cancer and its association with survival outcomes. It contributes to the understanding of this immune checkpoint, considering potential population-related and ethnic differences suggested in previous studies. We observed longer descriptive median progression-free survival and overall survival in PD-L1–positive patients; however, no statistically significant differences were observed in Kaplan–Meier survival curves by log-rank testing. PD-L1 positivity, measured using CPS, was associated with a lower risk of death in the adjusted Cox model. These findings should be interpreted cautiously and regarded as exploratory pending confirmation in larger studies.

## Data Availability

The research data are described in the article presented.
